# Levodopa Equivalent Dose of Safinamide: A Multicenter, Longitudinal, Case–Control Study

**DOI:** 10.1002/mdc3.13681

**Published:** 2023-02-15

**Authors:** Roberto Cilia, Emanuele Cereda, Marco Piatti, Andrea Pilotto, Luca Magistrelli, Nico Golfrè Andreasi, Salvatore Bonvegna, Elena Contaldi, Francesca Mancini, Gabriele Imbalzano, Rosa De Micco, Fabiana Colucci, Arianna Braccia, Gabriele Bellini, Francesco Brovelli, Roberta Zangaglia, Giulia Lazzeri, Maria Claudia Russillo, Enrica Olivola, Chiara Sorbera, Viviana Cereda, Patrizia Pinto, Patrizia Sucapane, Giorgio Gelosa, Mario Meloni, Francesca Pistoia, Maria Sessa, Margherita Canesi, Nicola Modugno, Claudio Pacchetti, Laura Brighina, Maria Teresa Pellecchia, Roberto Ceravolo, Mariachiara Sensi, Maurizio Zibetti, Cristoforo Comi, Alessandro Padovani, Anna L. Zecchinelli, Alessio Di Fonzo, Alessandro Tessitore, Francesca Morgante, Roberto Eleopra

**Affiliations:** ^1^ Department of Clinical Neurosciences, Parkinson and Movement Disorders Unit Fondazione IRCCS Istituto Neurologico Carlo Besta Milan Italy; ^2^ Clinical Nutrition and Dietetics Unit Fondazione IRCCS Policlinico San Matteo Pavia Italy; ^3^ Neurology Unit, Department of Neurology, Milan Center for Neuroscience San Gerardo Hospital Monza Italy; ^4^ Centro Parkinson e Parkinsonismi ASST Gaetano Pini‐CTO Milan Italy; ^5^ Neurology Unit, Department of Clinical and Experimental Sciences University of Brescia Brescia Italy; ^6^ Department of Translational Medicine Movement Disorders Centre, Neurology Unit, University of Piemonte Orientale Novara Italy; ^7^ IRCCS, Department of Neurology‐Stroke Unit and Laboratory of Neuroscience – Milan Istituto Auxologico Italiano Milan Italy; ^8^ Department of Neuroscience "Rita Levi Montalcini" University of Torino Turin Italy; ^9^ SC Neurologia 2U AOU Città della Salute e della Scienza Turin Italy; ^10^ Department of Advanced Medical and Surgical Sciences University of Campania “Luigi Vanvitelli” Naples Italy; ^11^ Azienda Ospedaliera Univerisitaria S. Anna, U.O. Neurologia Ferrara Italy; ^12^ University of Ferrara Ferrara Italy; ^13^ Unit of Neurology, Department of Clinical and Experimental Medicine University of Pisa Pisa Italy; ^14^ Parkinson's Disease and Movement Disorders Unit IRCCS Mondino Foundation Pavia Italy; ^15^ Neurology Unit, Department of Neuroscience Dino Ferrari Center, Fondazione IRCCS Ca’ Granda Ospedale Maggiore Policlinico Milan Italy; ^16^ Department of Medicine Surgery and Dentistry, Scuola Medica Salernitana, Neuroscience Section, University of Salerno Italy; ^17^ Parkinson and Movement Disorders Unit IRCCS Neuromed Pozzilli Italy; ^18^ IRCCS Centro Neurolesi “Bonino‐Pulejo” Messina Italy; ^19^ Department of Neurological Rehabilitation Parkinson's Disease and Movement Disorders Center, Moriggia‐Pelascini Hospital, Gravedona ed Uniti Gravedona Italy; ^20^ Neurology Unit ASST Papa Giovanni XXIII Bergamo Italy; ^21^ Neurology Unit San Salvatore Hospital L'Aquila Italy; ^22^ Neurology Unit ASST “Grande Ospedale Metropolitano” Niguarda Milan Italy; ^23^ IRCCS Fondazione Don Carlo Gnocchi ONLUS Milan Italy; ^24^ Department of Biotechnological and Applied Clinical Sciences University of L'Aquila L'Aquila Italy; ^25^ Neuroscience Research Centre Molecular and Clinical Sciences Institute, St. George's, University of London London UK; ^26^ Department of Clinical and Experimental Medicine University of Messina Messina Italy

**Keywords:** Parkinson's disease, levodopa equivalent dose, LED, safinamide, Rasagiline

## Abstract

**Background:**

Effects of dopaminergic medications used to treat Parkinson's disease (PD) may be compared with each other by using conversion factors, calculated as Levodopa equivalent dose (LED). However, current LED proposals on MAO‐B inhibitors (iMAO‐B) safinamide and rasagiline are still based on empirical approaches.

**Objectives:**

To estimate LED of safinamide 50 and 100 mg.

**Methods:**

In this multicenter, longitudinal, case–control study, we retrospectively reviewed clinical charts of 500 consecutive PD patients with motor complications and treated with (i) safinamide 100 mg (*N* = 130), safinamide 50 mg (*N* = 144), or rasagiline 1 mg (*N* = 97) for 9 ± 3 months and a control group of patients never treated with any iMAO‐B (*N* = 129).

**Results:**

Major baseline features (age, sex, disease duration and stage, severity of motor signs and motor complications) were similar among the groups. Patients on rasagiline had lower UPDRS‐II scores and Levodopa dose than control subjects. After a mean follow‐up of 8.8‐to‐10.1 months, patients on Safinamide 50 mg and 100 mg had lower UPDRS‐III and OFF‐related UPDRS‐IV scores than control subjects, who in turn had larger increase in total LED than the three iMAO‐B groups. After adjusting for age, disease duration, duration of follow‐up, baseline values and taking change in UPDRS‐III scores into account (sensitivity analysis), safinamide 100 mg corresponded to 125 mg LED, whereas safinamide 50 mg and rasagiline 1 mg equally corresponded to 100 mg LED.

**Conclusions:**

We used a rigorous approach to calculate LED of safinamide 50 and 100 mg. Large prospective pragmatic trials are needed to replicate our findings.

From 1990 to 2015, the prevalence of Parkinson's Disease (PD) doubled, and, keeping this similar growth rate, models of prediction estimate nearly 13 million people will be affected by 2040.^1^ Although no effective disease‐modifying therapy is available yet, the best medical treatment of PD patients consists of a combination of multiple medications acting synergistically to compensate for motor disability and improve patients’ quality of life.^2^

Although several drugs have been developed and marketed over the past two decades to provide better personalized therapy for PD patients,^3^ Levodopa remains the gold standard of symptomatic treatment. Currently, the total dose of dopaminergic therapy taken by a PD patient can be obtained by summing the Levodopa equivalent dose (LED) of different types of anti‐PD drugs, such as dopamine agonists, monoamine oxidase‐B inhibitors (iMAO‐B) and catechol‐O‐methyl transferase inhibitors, (iCOMT).^4^ LED conversion stemmed from the need to allow comparison of different treatment regimens in randomized clinical trials (RCTs) and has become increasingly useful in routine clinical practice to adjust patients’ therapy without inducing a negative effect on the overall clinical status.^4^, ^5^

Conversion factors may be used to switch from one dopaminergic drug to another within the same class (eg, dopamine agonists, iMAO‐B, iCOMT) or between different classes (eg, replacing a dopamine agonist with a iMAO‐B), or to allow compensatory increase of one drug while tapering another (eg, increasing Levodopa to reduce/withdraw dopamine agonist due to incident impulse control disorders or initiating device‐aided treatments).^5^ This minimizes the risk for either overdosing and causing medication‐induced side effects or underdosing with subsequent increase of OFF‐related disability.

Safinamide is a novel effective reversible iMAO‐B with both dopaminergic and nondopaminergic (including glutamate release modulation) mechanisms of action, that indicated as add‐on treatment to levodopa in fluctuating PD patients.^6^, ^7^ To date, there is no reliable information on LED of Safinamide at both 50 and 100 mg/day. It has been recently proposed that both safinamide 50 mg and safinamide 100 mg should be converted into 100 mg LED.^8^, ^9^ However, it has been clearly acknowledged that the major limitation of these proposed LED calculations so far is that they are “based on clinical experience and empirical approaches” without scientific and objective data, inclusive of their own proposal on safinamide.^8^

In the present study, we collected real‐life data on a large PD population to obtain a reliable calculation of LED of safinamide at a dose of 50 and 100 mg, as compared to control patients never treated with any iMAO‐B. In addition, we included a group of patients treated with rasagiline 1 mg, whose LED had been proposed to correspond to 100 mg despite the lack of data on dose equivalence,^4^ aiming to either confirm or update this conversion.

## Materials and Methods

### Patient Selection

We included patients who had received a clinical diagnosis of idiopathic PD^10^ and presented motor fluctuations and/or dyskinesias. We included subjects who received either (i) safinamide 100 mg, or (ii) safinamide 50 mg, or (iii) rasagiline 1 mg as add‐on therapy to levodopa for at least 6 months and had a follow‐up visit between 6 and 12 months (9 ± 3 months) after the initiation of iMAO‐B. As control group, we included (iv) patients with motor fluctuations and/or dyskinesias who had never been treated with any iMAO‐B.^11^ We excluded: (i) PD patients without motor complications, (ii) those on treatment with any iMAO‐B at baseline, or (iii) device‐aided therapies (deep brain stimulation or infusion therapies), (iv) atypical or secondary parkinsonism.

### Study Design

This retrospective, longitudinal, case–control study was conducted at 20 movement disorders centers throughout Italy. Movement disorders specialists at each participating center retrospectively reviewed demographic and clinical data from the electronic repositories from all consecutive PD patients visited between April 1, 2016, and October 31, 2019. Cases were excluded if the medical records did not contain well‐documented reports. General demographic (age, sex, body weight) and clinical data such as motor phenotype,^12^ age of onset, disease duration, Unified Parkinson Disease Rating Scale (UPDRS) from part I to part IV,^13^ and the Hoehn & Yahr stage that were already contained in clinical charts of patients were extracted and analyzed. In addition, items of UPDRS motor examination (Part III, collected in the ON‐medication state during the outpatient visit) were used to investigate dopaminergic and non‐dopaminergic deficiency scores, which indicate levodopa‐responsive features vs. axial impairment, respectively.^14^ Data on all PD therapies were obtained to calculate the number of daily Levodopa intakes, total Levodopa daily dose (mg/day), Levodopa dose adjusted for body weight (mg/kg/day) and for iCOMT, total LED from dopamine agonists (DA, mg/day) and the final total‐LED excluding iMAO‐B (mg/day).^4^ Data on amantadine and anticholinergics were collected. We additionally extracted from UPDRS parts I and II data on non‐levodopa‐responsive axial complications and UPDRS part IV subscores for dyskinesias and OFF periods.^15^ Anonymized patient data were extracted from medical records and recorded into an electronic case report form.

### Objectives

The primary objective was to estimate LED of safinamide 100 mg by calculating the difference in change at follow‐up in total LED between patients on this regimen and the control group. Secondary objectives included (i) to estimate LED of safinamide 50 mg and (ii) LED of rasagiline 1 mg in comparison to the control group; (iii) to investigate whether the use of iMAO‐B was associated with a reduction of concomitant PD medications; (iv) to compare among groups the difference in change at follow‐up of motor clinical variables according to the UPDRS.

### Ethics

The study was approved by the ethics committee of each participating center (coordinating center ethics committee: Neurological Institute Carlo Besta, Milan; CE n.68/2019) and conducted in accordance with the declaration of Helsinki and local regulatory requirements, including written informed consent to the use of patient anonymized clinical data for research purposes.

### Statistical Analysis

The sample size calculation was based on the primary endpoint (comparison of change in total LED from dopaminergic therapy between patients receiving safinamide 100 mg as add‐on therapy vs. patients receiving standard dopaminergic therapy without iMAO‐B medications). At baseline, it was expected a mean ± SD total LED of approximately 500 ± 350 mg/day.^11^ It has been calculated that at least 86 patients in each group will be required to detect a meaningful difference in the change of total LED at follow‐up. This was based on a statistical power of 90% [Type II error], a medium effect size of 0.5 and a two‐tailed test with a 5% significance level [Type I error]. Two additional groups of 86 subjects each were included, the former including patients treated with safinamide 50 mg/day (to calculate its LED, as secondary objective) and the latter including patients treated with rasagiline 1 mg (as active control product). Therefore, the minimum sample size was planned to be 344 patients.

Analyses were performed with the software STATA 15 or subsequent versions (StataCorp, College Station, TX, USA). Two‐tailed *p* values <0.05 will indicate statistical significance. Descriptive statistics of categorical variables are presented as counts and percentages, while continuous variables are reported as mean and standard deviation or median and inter‐quartile range [25th–75th percentile (inter‐quartile range, IQR)] according to the normality of distribution (checked using the Kolmogorov–Smirnov test). To minimize selection bias, all eligible patients were included consecutively without matching a priori for baseline characteristics; between‐group changes from baseline in continuous variables were analyzed using repeated‐measure linear regression model adjusted for disease duration, age at assessment, duration of follow‐up and the baseline value of each parameter. Huber‐White robust standard errors were used to account for study center.

A sensitivity analysis was conducted on patients showing stability in UPDRS‐Part III, defined as a change between the 25th and the 75th percentile of its distribution at follow‐up visit.

## Results

We collected data on a total population of 509 PD patients. Of these, six were excluded due to incomplete clinical data and three because of exclusion criteria (one was on selegiline at baseline and two had early PD with neither fluctuations nor dyskinesias). A total cohort of 500 patients was suitable for statistical analysis, distributed as follows: Safinamide 100 mg (*N* = 130), Safinamide 50 mg (*N* = 144), Rasagiline 1 mg (*N* = 97), and PD controls never treated with any iMAO‐B (*N* = 129).

Demographic and clinical data of the study population are shown in Table 1. At baseline, the four study groups had similar demographic (age, sex distribution, body weight) and clinical features (disease duration, severity of motor signs according to UPDRS‐III score and H&Y staging, motor phenotype, prevalence, and severity of motor complications according to UPDRS‐IV scores, namely OFF‐periods and Levodopa‐induced dyskinesias, LIDs). Compared to controls, patients on rasagiline had lower mean UPDRS‐II scores (*p* = 0.005) and marginally lower total LED (*p* = 0.046), mainly due to lower mean dose of Levodopa immediate release. There were no significant differences between the groups in non‐levodopa‐responsive motor complications.

**TABLE 1:** Clinical and treatment characteristics of the study population by use of Monoamine Oxidase type B Inhibitors

Variabl*e*	Safinamide 50 mg (*N* = 144)	Safinamide 100 mg (*N* = 130)	Rasagiline (*N* = 97)	No iMAO‐B (*N* = 129)	*P*‐value^a^
**Males**, *N* (%)	83 (57.6)	80 (61.5)	65 (67.0)	72 (55.8)	0.33
**Body weight**, (kg)	70.0 [12.2]	71.0 [12.7]	71.3 [11.0]	70.2 [12.3]	0.64
**Tremor‐dominant phenotype**, *N* (%), Mean [SD]	68 (47.2)	66 (50.8)	55 (56.7)	69 (53.5)	0.55
**Age at onset of disease** (years), Mean [SD]	59.6 [9.8]	58.5 [8.8]	59.5 [8.2]	60.1 [11.0]	0.42
**Age at assessment** (years), Mean [SD]	68.4 [9.4]	67.9 [9.0]	67.8 [8.1]	69.6 [8.9]	0.38
**Disease duration** (years), Mean [SD]	8.9 [4.8]	9.4 [4.9]	8.3 [5.0]	9.5 [5.2]	0.31
**UPDRS score** ^b^					
*Part I*, Mean [SD]	2.3 [2.4]	2.3 [2.3]	2.0 [1.9]	2.6 [2.1]	0.36
*Part II*, Mean [SD]	10.4 [6.2]	10.9 [7.0]	8.6 [6.5] †	12.9 [7.8]	**0.005**
*Part III*, Mean [SD]	21.9 [10.1]	22.8 [10.6]	21.2 [11.2]	22.0 [11.9]	0.58
*Part IV* (*motor complications*), Mean [SD]	3.8 [2.4]	4.1 [2.5]	3.4 [2.3]	3.9 [2.6]	0.11
**Dopaminergic deficiency score** ^c^, Mean [SD]	12.3 [6.6]	12.6 [6.6]	12.5 [7.2]	13.1 [7.6]	0.86
**Nondopaminergic deficiency score** ^c^, Mean [SD]	5.2 [3.1]	4.8 [3.2]	4.3 [3.3]	4.8 [3.4]	0.35
**Hoehn‐Yahr stage**, Mean [SD]	2.2 [0.7]	2.3 [0.6]	2.2 [0.6]	2.3 [0.7]	0.19
**Therapy**					
*Levodopa IR dose* (mg/day), Mean [SD]	560 [276]	534 [211] †	447 [295] †	618 [269]	**<0.001**
*Levodopa CR dose* (mg/day), Mean [SD]	26 [59]	54 [101]	38 [98]	35 [61]	0.12
*Number of daily Levodopa intakes*, Mean [SD]	4.6 [1.3]	4.8 [1.2]	3.9 [1.7] †	4.9 [1.8]	**<0.001**
*Total daily Levodopa dose* (mg/day), Mean [SD]	579 [287]	574 [205]	475 [304] †	644 [276]	**<0.001**
(mg/kg/day), Mean [SD]	8.3 [4.5]	8.1 [3.1]	6.9 [4.5] †	9.4 [4.4]	**0.002**
*Concomitant COMT inhibitors*, *N* (%)	32 (22.2)	24 (18.5)	15 (15.5)	26 (20.2)	0.61
*Levodopa dose adjusted for COMT inhibitors*					
(mg/day), Mean [SD]	620 [310]	608 [229]	546 [410] †	691 [317]	**0.041**
(mg/kg/day), Mean [SD]	8.9 [4.9] †	8.6 [3.6] †	7.9 [5.1] †	10.1 [4.9]	**0.002**
*Concomitant DA*, *N* (%)	95 (66.0)	86 (66.2)	62 (63.9)	76 (58.9)	0.55
*LED from DA* (mg/day), Mean [SD]	137 [130]	126 [121]	144 [145]	119 [130]	0.69
***Total‐LED*** (mg/day), Mean [SD]	757 [331]	734 [260]	691 [439] †	810 [348]	**0.046**
*Concomitant Amantadine*, *N* (%)	11 (7.6)	11 (8.5)	5 (5.2)	5 (3.9)	0.41
*Concomitant Anticholinergics*, *N* (%)	3 (2.1)	2 (1.5)	0 (0.0)	5 (3.9)	0.22
**Non‐levodopa‐responsive symptoms**					
*Dysphagia*, *N* (%)	9 (6.3)	8 (6.2)	5 (5.2)	14 (10.9)	0.31
*UPDRS‐ dysphagia*, Mean [SD]	0.3 [0.6]	0.4 [0.6]	0.2 [0.5]	0.4 [0.8]	0.56
*Frequent falls*, *N* (%)	21 (14.6)	15 (11.5)	7 (7.2)	20 (15.5)	0.24
*UPDRS‐ frequent falls*, Mean [SD]	0.6 [0.9]	0.5 [0.8]	0.3 [0.8]	0.6 [0.9]	0.11
*Freezing of gait*, *N* (%)	32 (22.2)	32 (24.6)	13 (13.4)	33 (25.6)	0.13
*UPDRS‐ freezing of gait*, Mean [SD]	0.6 [0.8]	0.7 [0.9]	0.4 [0.8]	0.8 [0.9]	0.080
*Postural instability*, *N* (%)	41 (28.5)	35 (26.9)	20 (20.6)	36 (27.9)	0.54
*UPDRS‐ postural instability*, Mean [SD]	0.9 [0.9]	0.8 [0.9]	0.7 [0.8]	0.9 [1.0]	0.41
**Motor complications**					
*Dyskinesias score*, Mean [SD]	1.3 [1.6]	1.2 [1.6]	0.9 [1.6]	1.2 [1.8]	0.24
*Dyskinesias, N* (%)	72 (50.0)	64 (49.2)	39 (40.2)	57 (44.2)	0.40
*OFF state*, Mean [SD]	2.3 [1.3]	2.7 [1.5]	2.3 [1.3]	2.5 [1.3]	0.39
*Fluctuations*, *N* (%)	126 (87.5)	123 (94.6)	84 (86.6)	115 (89.1)	0.16

Abbreviations: CR, Levodopa controlled release; COMT, catechol‐O‐methyltransferase; DA, dopamine agonists; iMAO‐B, MonoAmine Oxidase type B Inhibitors; IR, Levodopa immediate release; LED, levodopa equivalent dose; SD, standard deviation; UPDRS, Unified Parkinson's Disease Rating Scale.

^a^ According to parametric or non‐parametric analysis of variance (continuous variables; † significantly different from “No iMAO‐B group”) or Fisher's exact test (categorical variables) as appropriate.

^b^ In “ON” condition.

^c^ Calculated from UPDRS motor examination (part III) as proposed by Levy and colleagues.^13^

Changes in clinical features and pharmacological treatment at follow‐up are shown in Tables 2 and 3, respectively. After a mean follow‐up of 8.8‐to‐10.1 months, patients on safinamide 50 mg and 100 mg (but not those on rasagiline 1 mg) had lower UPDRS‐III scores than controls (*p* < 0.001), specifically concerning dopaminergic scores. This effect was greater with safinamide 100 mg compared to safinamide 50 mg, despite not reaching statistical significance. There were no significant changes in non‐dopaminergic motor features induced by iMAO‐B.

**TABLE 2:** Follow‐up clinical data (adjusted change) of the study population by use of Monoamine Oxidase type B Inhibitors

Variable	Safinamide 50 mg (*N* = 144)	Safinamide 100 mg (*N* = 130)	Rasagiline (*N* = 97)	No iMAO‐B (*N* = 129)	*P*‐value
**Follow‐up duration** (months), Mean [SD]	8.8 (3.9)	9.1 (3.7)	10.1 (4.8)	10.1 (3.6)	**0.016** ^a^
**Change in UPDRS score**					
*Part I*, Mean [SE]	−0.06 [0.11]	−0.04 [0.11]	+0.04 [0.13]	+0.12 [0.13]	0.67^c^
*Part II*, Mean [SE]	+0.33 [0.31]	−0.52 [0.30]	+0.19 [0.37]	+0.25 [0.36]	0.20^c^
*Part III*, Mean [SE]	−1.68 [0.50] †	−2.34 [0.53] †	−1.33 [0.61]	+0.88 [0.53]	**<0.001** ^c^
*Part IV* (*motor complications*), Mean [SE]	−0.30 [0.15]	−0.45 [0.15]	−0.44 [0.17]	−0.03 [0.16]	0.23^c^
**Dopaminergic deficiency score** ^b^, Mean [SE]	−0.41 [0.44] †	−0.92 [0.45] †	+0.11 [0.50]	+1.75 [0.41]	**<0.001** ^c^
**Nondopaminergic deficiency score** ^b^, Mean [SE]	+0.06 [0.17]	−0.02 [0.17]	+0.14 [0.19]	+0.12 [0.16]	0.92^c^
**Change in Hoehn‐Yahr stage**, Mean [SE]	+0.11 [0.03]	+0.04 [0.03]	+0.01 [0.04]	+0.03 [0.03]	0.11^c^
**Change in non‐levodopa‐responsive symptom score**					
*UPDRS‐ dysphagia*, Mean [SE]	+0.01 [0.04]	+0.01 [0.04]	+0.07 [0.05]	+0.12 [0.05]	0.62^c^
*UPDRS‐ frequent falls*, Mean [SE]	+0.05 [0.05]	−0.01 [0.05]	−0.03 [0.06]	+0.14 [0.06]	0.21^c^
*UPDRS‐ freezing of gait*, Mean [SE]	+0.06 [0.07]	+0.03 [0.06]	−0.05 [0.07]	+0.06 [0.07]	0.67^c^
*UPDRS‐ postural instability*, Mean [SE]	−0.01 [0.06]	+0.02 [0.06]	−0.1 [0.07]	+0.02 [0.06]	0.52^c^
**Change in motor complication score**					
*Dyskinesias*, Mean [SE]	−0.01 [0.10]	−0.01 [0.10]	+0.17 [0.11]	−0.02 [0.10]	0.55^c^
*OFF state*, Mean [SE]	−0.26 [0.10] †	−0.46 [0.10] †	−0.48 [0.11] †	−0.02 [0.10]	**0.006** ^c^

Abbreviations: COMT, catechol‐O‐methyltransferase; DA, dopamine agonists; iMAO‐B, Monoamine Oxidase type B Inhibitors; SD, standard deviation; SE, standard error; UPDRS, Unified Parkinson's Disease Rating Scale.

^a^ According to parametric analysis of variance.

^b^ Calculated from UPDRS motor examination (part III) as proposed by Levy and colleagues.^13^

^c^ According to mixed model for repeated measure analysis of variance († significantly different from “No iMAO‐B group”) adjusted for: disease duration, age at assessment, duration of follow‐up and the baseline value of each parameter.

^d^ According to Fisher's exact test.

**TABLE 3:** Data on pharmacological treatment at the end of follow‐up data of the study population by use of Monoamine Oxidase type B Inhibitors

Variable	Safinamide 50 mg (*N* = 144)	Safinamide 100 mg (*N* = 130)	Rasagiline (*N* = 97)	No iMAO‐B (*N* = 129)	*P*‐value
*Levodopa dose* (mg/day), Mean [SD]	583 [262] †	599 [236] †	523 [279] †	701 [237]	**<0.001** ^a^
(mg/kg/day), Mean [SD]	8.5 [4.2] †	8.4 [3.7] †	7.5 [4.2] †	10.2 [3.7]	**<0.001** ^a^
*Concomitant DA*, N (%)	86 (59.7)	80 (61.5)	58 (59.8)	80 (62.0)	0.97^b^
*Concomitant COMT inhibitors*, *N* (%)	27 (18.8)	19 (14.6)	14 (14.4)	33 (25.6)	0.085^a^
*Levodopa dose adjusted for COMT inhibitors*					
(mg/day), Mean [SD]	620 [276] †	625 [255] †	594 [406] †	764 [283]	**<0.001** ^a^
(mg/kg/day), Mean [SD]	8.9 [4.6] †	8.8 [4.1] †	8.7 [6.0] †	11.1 [4.4]	**<0.001** ^a^
*LED from DA* (mg/day), Mean [SD]	119 [131]	109 [115]	127 [145]	125 [133]	0.96^a^
*Total‐LED excluding iMAO‐B* (mg/day), Mean [SD]	740 [302] †	734 [280] †	686 [340] †	889 [316]	**<0.001** ^a^
**Change in therapy (crude)**					
*Levodopa dose* (mg/day), Mean [SD]	+4 [127]	+26 [149]	+48 [134]	+57 [180]	0.11^a^
*Levodopa dose* (mg/kg/day), Mean [SD]	+0.1 [1.7]	+0.3 [2.0]	+0.6 [2.3]	+0.8 [3.3]	0.31^a^
*Association of DA*	↓, N = 11; ↑, N = 2	↓, N = 10; ↑, N = 4	↓, N = 7; ↑, N = 3	↓, N = 6; ↑, N = 10	0.13^b^
*Association of COMT inhibitors*	↓, N = 5; ↑, N = 0	↓, N = 11; ↑, N = 5	↓, N = 4; ↑, N = 3	↓, N = 1; ↑, N = 8	**0.005** ^b^
*Association of Amantadine*	↓, N = 0; ↑, N = 2	↓, N = 0; ↑, N = 2	↓, N = 0; ↑, N = 0	↓, N = 0; ↑, N = 12	**<0.001** ^b^
*Association of Anticholinergics*	↓, N = 0; ↑, N = 0	↓, N = 0; ↑, N = 1	↓, N = 0; ↑, N = 0	↓, N = 0; ↑, N = 1	0.48^b^
*Levodopa dose adjusted for COMT inhibitors*					
(mg/day), Mean [SD]	0 [131] †	+17 [173] †	+48 [202]	+73 [174]	**0.002** ^a^
(mg/kg/day), Mean [SD]	+0.1 [1.8] †	+0.1 [2.4] †	+0.8 [3.5]	+1.0 [3.2]	**0.001** ^a^
*LED from DA* (mg/day), Mean [SD]	−18 [64] †	−16 [69] †	−17 [63] †	+6 [54]	**0.025** ^a^
***Total‐LED*** (mg/day), Mean [SD]	−17 [150] †	0 [162] †	−5 [208] †	+79 [174]	**<0.001** ^a^
**Change in therapy (Adjusted):**					
*Levodopa dose* (mg/day), Mean [SE]	+8 [15] †	+26 [14]	+27 [17]	+70 [15]	**0.037** ^c^
*Levodopa dose adjusted for COMT inhibitors*					
(mg/day), Mean [SE]	+4 [18] †	+18 [16] †	+33 [19] †	+83 [18]	**0.010** ^c^
(mg/kg/day), Mean [SE]	+0.2 [0.3] †	+0.1 [0.3] †	+0.5 [0.3] †	+1.2 [0.3]	**0.017** ^c^
*LED from DA* (mg/day), Mean [SE]	−17 [7]	−17 [7]	−14 [8]	+4 [7]	0.12^c^
***Total‐LED*** (mg/day), Mean [SE]	−13 [17] †	−2 [16] †	−17 [18] †	+91 [17]	**<0.001** ^c^
**SENSITIVITY ANALYSIS** ^a^					
**Change in therapy (Crude):**					
*Levodopa dose* (mg/day), Mean [SD]	+17 [115] †	+14 [153] †	+19 [156]	+73 [174]	**0.022** ^a^
*Levodopa dose adjusted for COMT inhibitors*					
(mg/day), Mean [SD]	+8 [122] †	−1 [169] †	+26 [247]	+80 [158]	0.**011** ^a^
*LED from DA* (mg/day), Mean [SD]	−20 [63]	−19 [89]	−12 [74]	+3 [56]	0.24^a^
***Total‐LED*** (mg/day), Mean [SD]	−12 [143] †	−20 [170] †	+14 [19]	+83 [152]	**0.023** ^a^
**Change in therapy (Adjusted):**					
*Levodopa dose* (mg/day), Mean [SE]	+26 [23]	+17 [20]	+48 [22]	+80 [21]	0.14^c^
*Levodopa dose adjusted for COMT inhibitors*					
(mg/day), Mean [SE]	+20 [26]	−2 [23]	+60 [25]	+86 [24]	**0.047** ^c^
*LED from DA* (mg/day), Mean [SE]	−21 [11]	−19 [10]	−9 [11]	+2 [10]	0.37^c^
***Total‐LED*** (mg/day), Mean [SE]^b^	−2 [25] †	−25 [21] †	0 [24] †	+94 [23]	**0.001** ^c^

Abbreviations: COMT, catechol‐O‐methyltransferase; DA, dopamine agonists; iMAO‐B, Monoamine Oxidase type B Inhibitors; LED, levodopa equivalent dose; SD, standard deviation; SE, standard error; UPDRS, Unified Parkinson's Disease Rating Scale.

^a^ Analysis conducted on patients reporting substantial stability in UPDRS‐Part III at follow‐up visit (*N* = 271) defined as a change between the 25th and the 75th percentile of its distribution (corresponding to −20% and + 15% of change): Safinamide 50 mg, *N* = 78; Safinamide 100 mg, *N* = 67; Rasagiline, *N* = 51; No iMAO‐B, *N* = 75.

^b^ Data used to calculate LED of Safinamide 100 mg, Safinamide 50 mg, and Rasagiline 1 mg (shown in Fig. 2).

^c^ According to parametric or non‐parametric analysis of variance († significantly different from “No iMAO‐B group”).

^d^ According to Fisher's exact test.

^e^ According to mixed model for repeated measure analysis of variance († significantly different from “No iMAO‐B group”) adjusted for: disease duration, age at assessment, duration of follow‐up and the baseline value of each parameter.

**FIG. 1:**
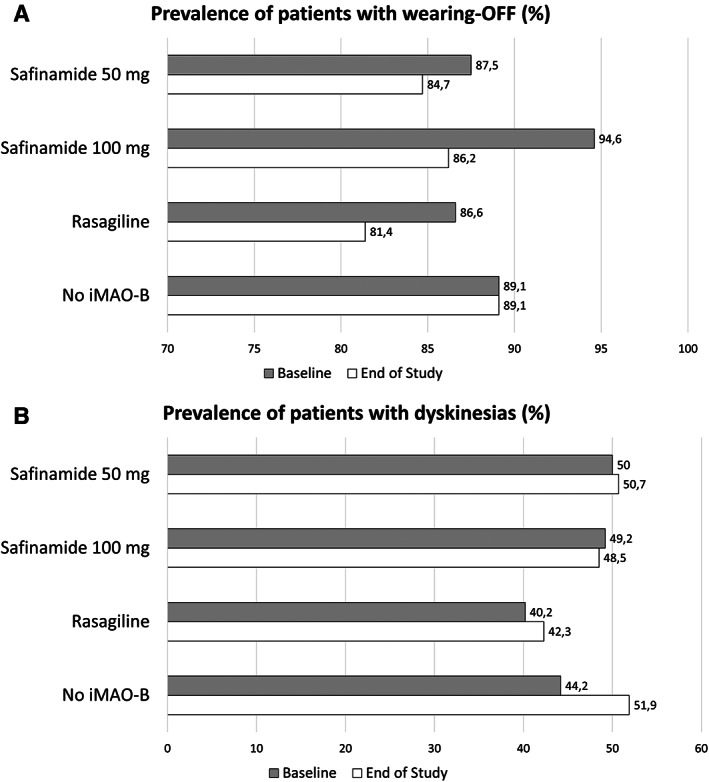
Prevalence of motor fluctuations (panel A) and dyskinesias (panel B) in the study population at baseline (gray color) and follow‐up (white color). No iMAO indicates the control group of patients who never received iMAO‐B.

Concerning motor complications, the three iMAO‐B groups had lower mean UPDRS‐IV scores related to OFF‐periods than controls and similar dyskinesias scores. The prevalence of patients complaining about OFF‐periods and LIDs (UPDRS‐IV OFF‐related items and LIDs items ≠ 0, respectively) showed similar, albeit nonsignificant, trends for lower prevalence of OFF‐periods in all iMAO‐B groups (Fig. 1A) and greater frequency of LIDs reported by control subjects (Fig. 1B). During follow‐up, patients in the control group without iMAO‐B had larger increase in total LED (*p* < 0.001) compared to the three iMAO‐B groups, particularly due to higher Levodopa dose. This was paralleled by a relative increase in new associations of iCOMT and amantadine in control subjects than the three iMAO‐B groups (Table 3). In particular, the use of safinamide 50 and 100 mg allowed to keep stable Levodopa dose adjusted for COMT inhibitors over time (significant difference compared to controls), whereas the rasagiline group did not differ from controls (Table 3). Direct comparison between safinamide 50 versus, 100 mg, safinamide 50 mg versus, rasagiline 1 mg, safinamide 50 mg versus, rasagiline 1 mg did not yield any significant difference.

### Sensitivity Analysis

Considering the significant effect played by the association of iMAO‐B on motor performance (as assessed by the UPDRS‐III, Table 2), a sensitivity analysis was conducted on patients reporting substantial stability in UPDRS‐Part III at follow‐up visit (*N* = 271), which was performed on the following groups: safinamide 50 mg, *N* = 78; safinamide 100 mg, *N* = 67; rasagiline, *N* = 51; control subjects, *N* = 75 (Table 3). Although crude sensitivity analysis showed larger effects of safinamide 50 and 100 mg (but not rasagiline) than control subjects on the dose of Levodopa immediate release and total LED, the adjusted analysis confirmed that total LED remained significantly lower in the three iMAO‐B groups than in the control group (*p* < 0.001, Table 3).

### LED Calculation

**FIG. 2:**
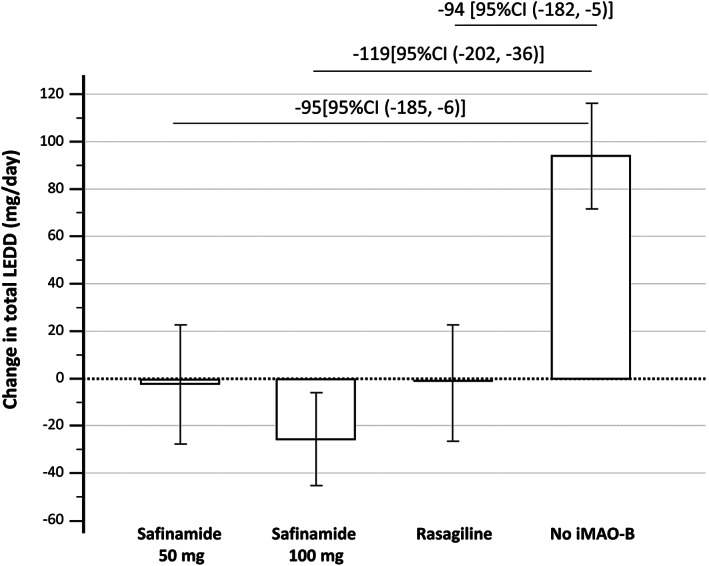
Adjusted mean difference [95%CI] in total LED change between each active group versus the control group used to calculate LED of Safinamide 50 mg, Safinamide 100 mg, and Rasagiline 1 mg. Each column represents the mean change in total LED between baseline and follow‐up for each study group, adjusted for disease duration, age at assessment, duration of follow‐up and the baseline value. No iMAO indicates the control group of patients who never received iMAO‐B.

According to our methodological approach to LED calculation using the mean difference in total LED change between each active group and the control group, data obtained from the primary analysis (after approximating by 6–8 mg/day) would be consistent with the conversion of all active groups to Levodopa 100 mg (Table 3). After adjusting for the effect of iMAO‐B on motor performance (sensitivity analysis), we obtained the following *conversion factors* (after approximating by 5–6 mg/day): Safinamide 100 mg = 1.25 (125 mg LED), Safinamide 50 mg = 2 (100 mg LED), Rasagiline 1 mg = 100 (100 mg LED) (Fig. 2, Table 3).

## Discussion

This multicenter study was specifically designed to calculate the conversion formula of LED of safinamide 50 and 100 mg on a large PD population, using a novel method that takes into account not only changes in dopaminergic medications (ie, total LED) but the clinical effects achieved. On the one hand, considering that total LED increases over time in PD patients with early fluctuations on medical therapy,^16^ our calculation of LED was based on the comparison between the change in total LEDD between each iMAO‐B group versus, control group, thus including not only the reduction of dopaminergic medications at follow‐up (as previous studies on LED) but also the therapy adjustment over time. On the other hand, our effort to provide an objective measure of LED included the observation that motor performance in the ON state (change in UPDRS‐III score in the ON‐medication state between baseline and follow‐up) may differ between different iMAO‐B type and dosage, reflecting a change in the dopaminergic boost. Accordingly, a formula predicting the longitudinal changes of levodopa dose requirements using real‐world UPDRS‐III scores has been recently proposed,^17^ confirming how LED and UPDRS‐III scores are closely related. It is worth mentioning that we designed a priori a short follow‐up observation period (9 ± 3 month) to minimize the confounding effect of disease progression on therapy adjustment and motor scores. In contrast with previous findings,^18^ we found that safinamide 50 and 100 mg provided a significant improvement of UPDRS‐III scores ON‐medication, basically due to reduced dopaminergic score.^14^ Why is this relevant? Let us consider an outpatient with suboptimal control of tremor and bradykinesia whose UPDRS‐III score is 18 with the drugs A + B and receives the add‐on drug C showing a 9‐point improvement of UPDRS‐III at follow‐up; the patient is satisfied and pharmacological therapy does not require any further change. How do we calculate the LED of C? Clearly, we cannot estimate it just by considering a reduction induced by C on total LED obtained from the A + B regimen, which did not occur in this case. As safinamide is an *add‐on* treatment for motor fluctuations, its association might not be followed by any change in concomitant medications at follow‐up. If we had not considered the change in UPDRS‐III by performing the sensitivity analysis, Safinamide 50 and 100 mg would have shared a similar 100 mg LED. Accordingly, we found a conversion factor of 1.25 for Safinamide 100 mg, which is 25% greater than the one currently used.^8^, ^9^ updating and overcoming the recent proposal to consider both safinamide 100 mg and 50 mg equal to Levodopa 100 mg despite the difference between the two dosages in terms of clinical effects,^7^, ^19^, ^20^ including MDS‐UPDRS‐III scores.^19^ Safinamide 50 mg and rasagiline 1 mg are equal to Levodopa 100 mg, which agrees with currently used estimates.^4^, ^8^, ^9^

Providing reliable LED conversion factors aims to minimize patient discomfort whenever major therapy adjustments are needed. To our opinion, these conversion formulae represent a step forward in literature on LED. First, our study overcomes the “*pseudo‐validity*” of all existing LED proposals, which are based on personal experience of individual neurologists and approaches far from being evidence‐based.^8^ Our study is an attempt to fill this gap and provide a framework for future studies aiming to provide objective measures of LED conversion formulae. Although previous RCTs on safinamide provided data on the relative changes in Levodopa dosage between the baseline and the end‐of‐study visits, none of them provided sufficient details on daily dose at baseline and/or on changes in other dopaminergic drugs (dopamine agonist and iCOMT) to allow any indirect inference on the conversion factor of safinamide.^7^, ^19^, ^20^, ^21^, ^22^, ^23^ Indeed, most studies limited their report on the relative number of patients on dopamine agonists and iCOMT neither reporting their LEDs at baseline nor their relative change at the end of the study. Second, it is worth highlighting that this is the first study supporting the conversion factor of 100 for rasagiline using an ad hoc study design. So far, rasagiline 1 mg has been considered equivalent to 100 mg Levodopa despite data on its dose equivalence had never been provided.^4^ In a previous 3‐year retrospective case–control study, the use of rasagiline was associated with a levodopa dose reduction of about 100 mg/day compared with patients who had never been treated with any MAO‐B inhibitor,^11^ indirectly supporting the present data.

Our findings provide useful information on the effects of iMAO‐B that are shared by safinamide and rasagiline. First, MAO‐B inhibition significantly reduced daily OFF periods without increasing LIDs, confirming data obtained from RCTs and meta‐analyses.^24^ Second, iMAO‐B provided evidence supporting their effectiveness in routine clinical practice. Indeed, their use is associated with some significant changes at follow‐up, such as (i) lower dose of levodopa‐based medications, (ii) lower OFF‐state frequency and severity, and (ii) an overall simplification of the therapeutic scheme, as reflected by the lower prescription of iCOMT and amantadine at follow‐up compared to control subjects, thus reducing the cumulative risk and severity of motor complications as well as adverse events. It should be noted that the similar severity of dyskinesias between those on iMAO‐B and controls might have been masked by the relative increase of amantadine use in the control group.

There are limitations to acknowledge. The retrospective nature of the study intrinsically harbors potential prescription bias, such as the preference of clinicians to keep a simplified therapeutic regimen without iMAO‐B in patients with psychosis and the slightly lower UPDRS‐II scores and total LED at baseline in patients on rasagiline than control subjects. However, it is unlikely that these minor differences played a confounding effect of the results, because (i) the four groups had similar major demographic and clinical features (age, sex, motor phenotype, disease duration and severity) and (ii) all analyses were adjusted for several potential confounders, such as disease duration, age at assessment, duration of follow‐up and the baseline value of each parameter. Nevertheless, prospective pragmatic real‐world clinical trials on large cohorts of PD patients with early motor fluctuations are warranted to replicate our results. On the other hand, this design may also be considered a strength of the study as it allowed us to collect real‐life data on consecutive patients that is relatively less biased than the data obtained from more homogeneous but selected cohorts reported in clinical trials. Another strength is the large population of 500 patients recruited by neurologists with heterogeneous prescription patterns from 20 movement disorders clinics throughout Italy, which further increase the generalizability of our results.

In conclusion, according to the results of the present study, we propose that safinamide 100 mg corresponds to 125 mg of Levodopa, whereas safinamide 50 mg and rasagiline 1 mg equally correspond to 100 mg of Levodopa. Future studies aiming to define LED of dopaminergic drugs should apply rigorous methods and use real‐life data on a large PD population.

## Author Roles

(1) Research project: A. Conception, B. Organization, C. Execution; (2) Statistical Analysis: A. Design, B. Execution, C. Review and Critique; (3) Manuscript Preparation: A. Writing of the first draft, B. Review and Critique.

R.C.: 1A, 1B, 1C, 2A, 2C, 3A, 3B.

E.C.: 1A, 2A, 2B, 3B.

M.P.: 1C.

A.P.: 1C, 3B.

L.M.: 1C.

N.G.A.:1C, 3B.

S.B.: 1C.

E.C.: 1C.

F.M.: 1C, 3B.

G.I.: 1C.

R.D.M.: 1C, 3B.

F.C.: 1C, 3A.

A.B.: 1C.

G.B.: 1C.

F.B.: 1C.

R.Z.: 1C.

G.L.: 1C, 3B.

M.C.R.: 1C.

E.O.: 1C.

C.S.: 1C.

V.C.: 1C.

P.P.: 1C.

P.S.: 1C.

G.G.: 1C.

M.M.: 1C, 3B.

F.P.: 1C, 3B.

M.S.: 1B.

M.C.: 1B.

N.M.: 1B.

C.P.: 1B.

L.B.: 1B.

M.T.P: 1B, 3B.

R.C.: 1C, 3B.

M.C.S.: 1C, 3B.

M.Z.: 1B, 3B.

C.C.: 1B.

A.P.: 1B.

A.L.Z.: 1B.

A.D.F.: 1B, 3B.

A.T.: 1B, 3B.

F.M.: 1B, 3B.

R.E.: 1B, 3B.

## Disclosures

**Ethical Compliance Statement:** We confirm that we have read the Journal's position on issues involved in ethical publication and affirm that this work is consistent with those guidelines. Ethics Committee of the coordinating center: Fondazione IRCCS IStituto Neurologico Carlo Besta, Milano; reference number: CE n.68/2019. The study was approved by the ethics committee of each participating center and conducted in accordance with the declaration of Helsinki and local regulatory requirements, including written informed consent to the use of patient anonymized clinical data for research purposes.

**Funding Sources and Conflicts of Interest:** All the authors report no conflict of interest related to this manuscript.

**Financial Disclosures for the Previous 12 Months:** RCil has received speaking honoraria from Zambon Italia; Zambon SAU; Bial Italia Srl; Advisory board fees from Bial; Research support from the Italian Ministry of Health; Editor‐in‐chief of the neuromuscular and movement disorders section of Brain Sciences; Member of the editorial board of Parkinsonism and related disorders and Frontiers in Neurology. EC has received speaking honoraria from Zambon Italia. APil is supported by IMI H2020 initiative (IMI2‐2018‐15‐06) paid to the university of Brescia Italian Ministry ofHealth; he received lecture honoraria from Bial, Biomarin, Abbvie, Chiesi, Roche and Zambon Italia (payments made to AP as an individual); he received research support from Bial, Biomarin, Abbvie, Chiesi, and Zambon pharmaceuticals (payment made to the Institution University of Brescia). FP has received speaking honoraria from Novartis. MTP has received compensation for consultancies from Zambon Italia, Theravance, Teva, Orion. RCer has received speaking honoraria from Zambon Italia, Abbvie, Lusofarmaco, General Electric. MZ has received speaking honoraria from Medtronic, Bial, and AbbVie. APad is consultant and served on the scientific advisory board of GE Healthcare, Eli‐Lilly and Actelion Ltd. Pharmaceuticals and received speaker honoraria from Nutricia, PIAM, Langstone Technology, GE. Healthcare, Lilly, UCB Pharma and Chiesi Pharmaceuticals. He is funded by grant of the Ministry of University (MURST). FMor has received speaking honoraria from Abbvie, Medtronic, Zambon. SpA, Bial, Merz; Travel grants from the International Parkinson's disease and Movement Disorders. Society; Advisory board fees from Merz; Consultancies fees from Merz and Bial; Research support from Boston Scientific Merz and Global Kinetic; Royalties for the book “Disorders of Movement” from Springer; member of the editorial board of Movement Disorders, Movement Disorders. Clinical Practice, European Journal of Neurology. All other authors report no financial disclosures.

## Data Availability

The data that support the findings of this study are available from the corresponding author upon reasonable request.
